# Nonparametric approaches for population structure analysis

**DOI:** 10.1186/s40246-018-0156-4

**Published:** 2018-05-09

**Authors:** Luluah Alhusain, Alaaeldin M. Hafez

**Affiliations:** College of Computer and Information Sciences, King Saud University, Riyadh, Saudi Arabia

**Keywords:** Population structure analysis, Clustering, Dimension reduction, Principal component analysis, Allele-sharing distance, Genetic data, Single nucleotide polymorphism, Population genetics

## Abstract

The analysis of population structure has many applications in medical and population genetic research. Such analysis is used to provide clear insight into the underlying genetic population substructure and is a crucial prerequisite for any analysis of genetic data. The analysis involves grouping individuals into subpopulations based on shared genetic variations. The most widely used markers to study the variation of DNA sequences between populations are single nucleotide polymorphisms. Data preprocessing is a necessary step to assess the quality of the data and to determine which markers or individuals can reasonably be included in the analysis. After preprocessing, several methods can be utilized to uncover population substructure, which can be categorized into two broad approaches: parametric and nonparametric. Parametric approaches use statistical models to infer population structure and assign individuals into subpopulations. However, these approaches suffer from many drawbacks that make them impractical for large datasets. In contrast, nonparametric approaches do not suffer from these drawbacks, making them more viable than parametric approaches for analyzing large datasets. Consequently, nonparametric approaches are increasingly used to reveal population substructure. Thus, this paper reviews and discusses the nonparametric approaches that are available for population structure analysis along with some implications to resolve challenges.

## Background

Population structure analysis is a major area of interest within the field of genetics and bioinformatics. Population structure is the grouping of individuals into subpopulations based on observable characteristics, such as culture, language, geographical region, and physical appearance [1]. Since patterns of genetic variation exist among people, genetic research is concerned with characterizing the genetic variations of populations and summarizing the relationships between individuals from genetic data. Thus, the analysis of population structure involves the identification of shared genetic variations among individuals and, accordingly, the grouping of similar individuals into subpopulations.

The inference of population structure from genetic markers is very helpful in different applications, such as genome-wide association studies (GWAS) [2–8] and forensics [9]. In GWAS, case-control studies aim to scan a large portion of the genome to identify the responsible genes for different diseases via associations between a genetic marker and a disease. The presence of population structure might result in spurious associations between a marker and a disease, which occur when most of the samples in the case group are from a specific population. Subsequently, a marker appears significantly more frequently in the case than in the control group, so this marker is incorrectly considered to be associated with the disease. Consequently, inferring population structure is a prerequisite for association mapping studies to avoid making spurious correlations or missing genuine correlations, which would eventually reduce false positive rates. In forensics, identifying population substructure is a prerequisite for developing reference panels. Reference panels are composed of a set of genetic markers that can provide information on an individual’s ancestry [10].

Populations are genetically structured into distinct subpopulations [11]. Thus, the main research question is how to assign *n* individuals using *m* genetic markers to *K* subpopulations. Therefore, research in population structure addresses the following problems: how to detect population structure, how to assign individuals to their corresponding subpopulation, how to determine the optimal number of subpopulations, how to reduce the number of genetic markers needed for inference of population structure, how to infer population structure at a fine scale, and finally, how to handle large genetic datasets [11–16].

Several methods can be utilized to uncover population substructure. In general, these methods can be categorized into two broad approaches: parametric and nonparametric. Parametric approaches use statistical models to infer population structure and assign individuals into subpopulations. However, these approaches suffer from many drawbacks that make them impractical for large datasets. Such drawbacks include an intensive computational cost, genetic assumptions that must be held, and sensitivity to sample size. In contrast, nonparametric approaches have the advantage of efficient computational cost and no modeling assumption requirements, making them more viable than parametric approaches for analyzing large datasets.

**Fig. 1:**
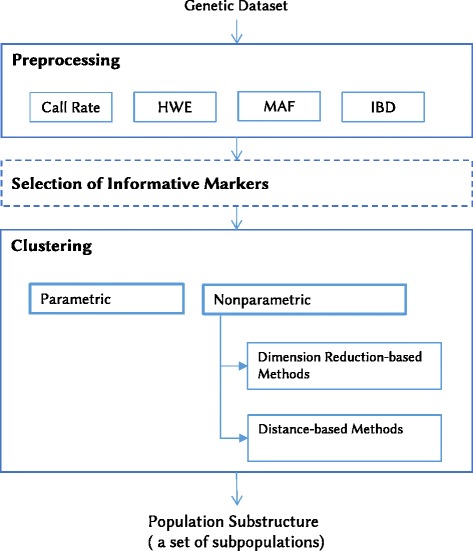
A general workflow for population structure analysis

Advances in DNA sequencing technology have provided genome-wide single nucleotide polymorphisms (SNPs) that have enabled the study of genetic variation at an unprecedented resolution. Detailed characterization of genetic variations across all chromosomes is possible using thousands of markers spanning the entire genome. Consequently, nonparametric approaches are increasingly being used to reveal population structure because of their great advantage of efficiency in handling high-dimensional genetic datasets. Therefore, this paper reviews the literature on the topic of population structure analysis with an emphasis on nonparametric approaches. The purpose of this paper is to review the nonparametric methods available to infer population structure from genetic data. The paper comprises seven sections, including this background section. It begins by outlining the background information required to understand the genetic data used for the analysis, along with the data preprocessing. Then, an overview of the parametric and nonparametric approaches of population structure analysis is presented. Since nonparametric approaches are more viable than parametric approaches for analyzing large datasets, this paper is concentrated on the nonparametric approaches proposed to address the inference of population structure from genetic data. These approaches are categorized into dimension reduction-based methods and distance-based methods. Afterward, the paper discusses the literature on the selection of informative markers. Finally, the paper concludes with a comprehensive discussion of the literature. Figure 1 provides a general workflow for population structure analysis, where the input is the genetic dataset and the output is the population substructure as a set of subpopulations (i.e., clusters).

## Genetic data

### Data description

The most widely used markers to study the variation of DNA sequences are SNPs [17]. SNPs take the form of substitutions at a single base pair. An SNP occurs when a single nucleotide from a DNA sequence differs at the same position between individuals. Since SNPs arise in certain populations only, they are very useful to differentiate and analyze different populations. In practice, genotyping is an inexpensive process used to examine DNA samples to determine which alleles appear in particular loci. Therefore, genotyping produces a genotypic profile of an individual as an unordered set of alleles that appears at each locus. In this profile, the nucleotides are encoded as two alleles, allele (A) and allele (B). Therefore, three distinct genotypes can appear at a locus: wild-type homozygous (AA), homozygous (BB), and heterozygous (AB). Nevertheless, an SNP marker can be encoded as 0, 1, or 2 according to the number of reference alleles. Thus, it has the advantage of being handled as a numerical variable that represents the number of reference alleles.

Many datasets are available online to study population structure. These datasets consist of genotyped markers along with information about individuals, where the population label is the most required information for population structure analysis. The most well-known datasets are HapMap [18–20], 1000 Genomes Project [21], and Pan-Asian [22].

### Data preprocessing

The preprocessing of genetic data is a necessary step to examine the quality of data and determine which markers or individuals can reasonably be included in the analysis [23]. First, the quality of the SNP markers is assessed, including the following:

- SNP call rate: SNP call rate is assessed to verify the amount of missing data for each marker. SNP call rate is the proportion of genotypes per marker with non-missing data. Usually, a threshold of 95% is used to remove these poorly genotyped SNPs. However, the threshold should be set carefully to avoid removing important markers.
- Hardy-Weinberg equilibrium (HWE): HWE [12] verifies the assumptions of Hardy-Weinberg. So, a statistical test is applied to determine whether a marker follows the Hardy-Weinberg equilibrium or not. If a marker deviates from the equilibrium, then it may be because of genotyping errors; therefore, it should be excluded.
- Minor allele frequency (MAF): MAF denotes the frequency of a marker’s less frequent allele in a given population. SNPs with low MAF should be excluded, and a threshold of 1–2% is typically applied.

For this assessment, PLINK [24] is typically used to prune SNPs with a minor allele frequency greater than 5%, a missing rate less than 5%, and a Hardy-Weinberg equilibrium (HWE) deviation *p* value of no less than 0.05.

Then, an assessment is performed to check the quality of the individuals, which includes the following:

- Individual call rate: Individual call rate refers to the proportion of genotypes per individual with non-missing data. The missingness rate should not exceed a certain threshold.
- Identity by descent (IBD): IBD [25] is calculated to assess which individuals are related. It indicates whether a pair of individuals has identical copies of the same ancestral allele. The proportion of shared alleles between a pair of individuals determines the relation between them, such as identical twins, first-degree relatives (i.e., full siblings, parent–offspring), second-degree relatives (i.e., half-siblings, uncle/aunt, nephew/niece), and third-degree relatives (i.e., cousins). Related individuals are excluded. In practice, relatedness can be assessed using kinship coefficients estimated by KING [26]. The KING command can be used to filter out related individuals, where a threshold of a degree relationship can be specified.

## Parametric approaches

Parametric approaches use statistical models to infer population structure and assign individuals into subpopulations. These models are used to estimate population parameters, such as allele frequency, for the population and to calculate the likelihood that an individual belongs to a specific subpopulation [12, 27]. Parametric approaches are based on several genetic assumptions about the data, including the Hardy-Weinberg equilibrium (HWE) [12] for populations and the linkage equilibrium (LE) [28] between loci within each population.

Essentially, a parametric approach infers ancestral proportions for each individual and then groups individuals who have similar patterns of inferred ancestry [16]. The majority of parametric methods for population structure analysis apply Bayesian inference. Bayesian inference is applied to model the probability of observed genotypes given the individual ancestry proportions and population allele frequencies. These methods simultaneously assign individuals to populations and identify populations from genotype data based on the estimation of the allele frequencies for each population [13, 29, 30].

STRUCTURE is a widely used parametric method that relies on Bayesian MCMC [12, 29]. In particular, Markov chain Monte Carlo (MCMC) based on Gibbs sampling is implemented to estimate the posterior distribution of allele frequency given the probability of ancestral populations of individuals and allele frequencies for all populations. Similar to STRUCTURE, PARTITION [31], BAPS/BAPS2 [32, 33], and GENELAND [34] take the same modeling approach, which is based on an MCMC algorithm, to sample the posterior distribution. Moreover, FRAPPE [35] and ADMIXTURE [30, 36] adopt the same modeling approach but rely on maximizing the likelihood using an expectation-maximization (EM) algorithm instead of sampling the posterior distribution. In contrast, L-POP [27] implements a maximum likelihood approach based on latent class analysis (LSA), whereas PSMIX [37] uses the same approach via the implementation of a mixture model. Recently, fast STRUCTURE [38] was developed to improve the inference model underlying STRUCTURE using a variational Bayesian method. Variational methods optimize the computation of posterior distributions and accelerate the inference process.

Parametric approaches estimate the observed allele frequency for each population using statistical inference models that include some parameters and are based on probability distribution. Before running these methods, parameters must be set, such as the number of populations *K*, the most critical parameter. Accordingly, a parametric approach suffers from many drawbacks: First and most importantly, the intensive computational cost makes it impractical for large-scale datasets containing thousands of individuals and thousands of markers [39–41]. Second, parametric approaches are developed on the basis of the genetic assumptions of the Hardy-Weinberg equilibrium (HWE) and the linkage equilibrium (LE) between loci within each population. As a result, they can be very misleading when data assumptions cannot be verified or are invalidated [35, 40]. In specific, LE does not hold when a vast amount of genetic data are used [42]. Third, parametric methods depend on an estimation of allele frequency that is sensitive to sample size. Consequently, allele frequency is subject to high variations when using small samples representing each subpopulation [29, 42]. Lastly, parametric methods are not applicable to analyzing large and highly structured population datasets because of the limited number of *K* clusters that can be inferred [16].

## Nonparametric approaches

Nonparametric approaches have been proposed to address the problem of analyzing population structure from genetic data in order to overcome the drawbacks of parametric approaches. Nonparametric approaches group individuals with similar genetic profiles together [16]. In 2006, Liu and Zhao [40] proposed a two-stage nonparametric strategy for analyzing population structure from genetic data with the goal of facilitating the clustering process of the high-dimensional space of genotype data. The first stage involves reducing the dimensionality of the genotypic dataset using multivariate analysis methods, such as singular value decomposition (SVD) and principal component analysis (PCA). The second stage involves applying clustering algorithms to identify population substructure from the reduced data. Another nonparametric strategy is to calculate the pairwise distances between individuals and then perform clustering. Both strategies have the advantage of identifying a population structure and assigning individuals to their corresponding subpopulation. Indeed, both strategies provide a framework for population structure analysis from genetic data where different methods can fit into that framework.

Nonparametric approaches have many advantages, including an efficient computational cost and no modeling assumption requirements. Nonparametric approaches have a more efficient computational cost compared to parametric approaches, making the former more viable for analyzing large datasets [15]. Also, nonparametric approaches do not make any assumption on genetic data, which is a great advantage over parametric approaches [43]. Therefore, when a large amount of genotype data is available, nonparametric approaches are preferred, as there is no need to verify the assumptions of Hardy-Weinberg and the linkage equilibrium [44]. Moreover, since these approaches are not dependent on estimating allele frequencies, they are unaffected when the number of individuals representing a subpopulation is small [42].

There are many nonparametric methods. Some methods use a dimension reduction technique to reduce the dimensions of genetic markers before conducting a clustering. Other methods consider computing dis/similarity matrices of the data where a clustering technique is applied. Thus, nonparametric methods can be categorized into dimension reduction-based methods and distance-based methods.

### Dimension reduction-based methods

Dimension reduction-based methods are based on mapping high-dimensional genetic data to low-dimensional space and then applying clustering on the reduced dimensions. Principal component analysis (PCA) is the most cited dimension reduction method used to detect population structure based on genetic data [45, 46]. Typically, PCA’s scatterplots are used to visualize population structure, where the most genetically isolated subpopulations appear as distinct clusters of individuals. Most importantly, PCA can be used to infer spatial population genetic variations [47].

EIGENSTRAT\smartpca [39, 41] is the most used PCA-based tool for detecting population structure. In EIGENSTRAT\smartpca, eigenanalysis is used to detect population substructure, such that eigenvalues and eigenvectors capture the amount and axes of variation among individuals, respectively. Thus, the principal components (PCs), or eigenvectors, serve as the new reduced dimensions. Similar to EIGENSTRAT\smartpca, PLINK [24] and SNPRelate [48] can be used to apply PCA on genetic datasets.

#### Principal components analysis

Given $$ x={\left({x}_{i,l}\right)}_{,\begin{array}{c}1\le i\le n\\ {}1\le l\le p\end{array}} $$ is an *n* × *p* matrix, where *n* is the number of individuals and *p* is the number of SNPs. Each entry *x*_*i*, *l*_ corresponds to the genotype of individual *i* for the marker *l*, coded as 0, 1, or 2 according to the number of reference alleles present at the locus *l*.

To perform a principal components analysis (PCA) on the matrix *x*, the data are first centered and normalized. The column means *μ*_*l*_ and the observed allele frequency of each marker *p*_*l*_ are computed as follows:

$$ {\mu}_l=\frac{\sum_{i=1}^n{x}_{il}}{n} $$

$$ {p}_l=\frac{1+{\sum}_{i=1}^n{x}_{il}\ }{2+2\ n} $$

The new genotype matrix $\overset{\sim }{x}$is defined, such that each entry is:

$$ {\overset{\sim }{x}}_{il\kern0.5em }=\frac{x_{il}-{\mu}_l}{\sqrt{p_l\left(1-{p}_l\right)}} $$

Based on the *n* × *n* covariance matrix, a singular vector decomposition is computed as:

$$ \frac{1}{p}\ \overset{\sim }{x\ }\ {\left(\overset{\sim }{x\ }\ \right)}^T $$

Then, a set of principal components (*PC*_1_, *PC*_2_, …, *PC*_*n* − 1_) are generated [41, 49].

A major issue with PCA applied to genetic data is how to determine the number of significant principal components, which is the number of principal components needed to sufficiently describe a structure of the population [13]. The EIGENSTRAT algorithm applies a variant of eigenanalysis to determine the significant principal components based on Tracy-Widom (TW) theory [50]. TW theory states that the distribution of the largest eigenvalue approximately follows the TW distribution when the dimension of a matrix is suitably large [51]. Hence, the TW distribution is used to determine the probability of population substructure.

Principal components can be used as the axes of variations to provide a graphical overview of the population structure. This graphical representation of the individuals can highlight outlier individuals, or those which seem to lie farther out than the others. Also, the set of significant principal components can be used to cluster individuals into genetically homogeneous subpopulations. For instance, the Gaussian mixture model or *K*-means algorithm can be applied to these principal components [52].

#### Clustering based on principal components

Different clustering algorithms can be applied to the principal components. Since the principal components are normally distributed, they fit well with the Gaussian mixture model (GMM) clustering. Therefore, the PCAclust algorithm [52] was proposed as three steps. The first step involves applying PCA to the genetic data to compute the principal components (PCs). Then, a set of significant PCs is selected using the TW statistic at a 5% level. Finally, the selected PCs are clustered using the GMM algorithm to group the individuals into populations.

Moreover, Lee et al. [52] have proposed using PCA for dimension reduction with three clustering algorithms: *K*-means [53], the mixture model [54], and spectral clustering [55]. They used Gap statistics [56] and the Bayesian information criterion (BIC) [57] to predict the optimal number of clusters. In their experiment, they showed that all three algorithms have comparable results. However, the different clustering algorithms showed different degrees of sensitivity to noisy and non-informative markers, which demonstrated the importance of selecting a proper set of informative markers.

Furthermore, iterative pruning PCA (ipPCA) was proposed to resolve the highly structured population that appears as a conglomerate in PCA space. ipPCA does this by iteratively applying PCA to decompose the structure of the population. The ipPCA method has two versions, TW-ipPCA [11] and EigenDev-ipPCA, [16], which differ in their termination tests. Recently, HiClust-ipPCA [58] was proposed as a variation of EigenDev-ipPCA wherein hierarchical clustering is used.

The PCA-based ipPCA method [11] has been proposed to address the overlapping problem that appears in PCA space when analyzing closely related subpopulations. The ipPCA method can detect population structure at a fine scale by iteratively bisecting individuals based on a termination test that checks whether a significant structure is present. In ipPCA, PCA is applied, and then a termination test is verified to decide whether to advance to clustering or to stop. Clustering is performed based on significant PCs. The number of significant PCs depends on the number of individuals in the dataset, such that later iterations require fewer PCs for clustering than earlier iterations. Therefore, the new bisected datasets will have fewer individuals. ipPCA iterates until all individuals have been assigned to homogeneous subpopulations. At the end, the number of subpopulations *K* is determined by counting all the terminal nodes or subpopulations. ipPCA uses a fuzzy C-mean algorithm to split the dataset into two parts. Indeed, the iterative pruning nature of ipPCA offers a logical way to present the degree of relatedness between subpopulations.

ipPCA has two different versions: TW-ipPCA [11] and EigenDev-ipPCA [16]. TW-ipPCA applies the TW test as a termination criterion [41]. TW, as previously mentioned, is implemented in the EIGENSTRAT/smartpca algorithm for detecting whether a significant structure is present in the dataset. TW-ipPCA suffers from type 1 error when the sample size is large, and subsequently, a group of individuals belonging to a single subpopulation would be assigned into separate subpopulations.

EigenDev-ipPCA was proposed to address the spurious cluster problem using a heuristic called EigenDev as a termination criterion [16]. EigenDev is inspired by the Eigenvalue Grads heuristic [59], which is applied in the signal processing domain. The EigenDev statistic is based on the eigenvalues of the data matrix; it has no hidden parameters and is more robust to type 1 error. The application of EigenDev to ipPCA improves the accuracy of individuals’ assignments and the estimation of the number of subpopulations, especially when using huge and complex datasets. EigenDev-ipPCA reveals subpopulations that are subclusters of subpopulations generated by TW-ipPCA.

HiClust-ipPCA [58] is another variation of ipPCA that employs hierarchical clustering instead of fuzzy C-mean within the ipPCA framework. In addition, a PCA-based feature selection is applied as a data preprocessing step. In each iteration, PCA is applied to select the most informative markers. Then, PCA is applied to the selected markers to map them to a reduced space. Next, a hierarchical clustering with Ward’s minimum variance is applied to cluster data into two groups. This process is iterated until satisfying a termination condition. The experiments illustrate that hierarchical clustering provides better clustering results than fuzzy C-mean and that the use of the feature selection technique is effective for reducing data dimensions and increasing computational efficiency.

#### Other dimension reduction methods

There are many alternatives to PCA, such as singular value decomposition (SVD) [60]. Liu and Zhao [40] used SVD for dimension reduction and density-based mean clustering (DBMC) for clustering. SVD is used because it is efficient for a large matrix of markers and individuals. DBMC was proposed as a variant of *K*-means that can determine the number of clusters automatically, because *K*-means requires the number of clusters to be given. The similarity between individuals is measured using Cosine similarity. The performance of DBMC was compared with *K*-means and the mixture model [40], and it was found that the mixture model and DBMC performed better than *K-*means. Another alternative of PCA is multi-dimensional scaling (MDS), which uses a similarity matrix between the individuals instead of the data matrix to create axes of variation [61].

Table 1 describes the nonparametric dimension reduction-based methods in terms of dimension reduction and/or proximity measure, clustering technique, and the package/tool if it is available.

**Table 1:** Dimension reduction-based methods of population structure analysis

Reference	Dimension reduction	Distance matrix	Clustering	Tool/package
Patterson at el. (2006) [41]	PCA (TW)	–	–	EIGENSTRAT/smartpca [82]: Perl
Liu at el. (2006) [40]	SVD	Cosine similarity	Density-based mean clustering (DBMC)	–
Lee at el.(2009) [52]	PCA (TW)	–	Spectral clustering (*K*-means, mixture model)	–
Intarapanich at el. (2009) [11]	PCA (TW)	Euclidean distance	Fuzzy C-means	TW-ipPCA [83]: MATLAB
Limpiti at el.(2011) [16]	PCA (EigenDev)	Euclidean distance	Fuzzy C-means	EigenDev-ipPCA [83]: MATLAB
Amornbunchornvej at el. (2012) [58]	PCA	ASD	Ward’s clustering	–

### Distance-based methods

Distance-based methods are based on computing the pairwise similarities/distances between individuals. The allele-sharing distance (ASD) [44, 62] is a measure proposed for determining the genetic proximity between each pair of individuals. Distance-based methods usually apply a clustering on the ASD matrix to infer population structure. For instance, allele-sharing distance and Ward’s minimum variance hierarchical clustering (AWclust) [42, 44] applies an agglomerative hierarchical clustering to ASD, while Spectral Hierarchical clustering for the Inference of Population Structure (SHIPS) [43] uses divisive clustering. Furthermore, NETVIEW [63] reveals the hierarchy of population substructures based on a representation of the genetic data as a network of individuals connected by edges representing the ASD between each pair. Iterative neighbor-joining tree clustering (iNJclust) [64] performs a graph-based clustering on a neighbor-joining (NJ) tree. Table 2 describes the distance-based methods in terms of the proximity measure, clustering technique, and available package/tool.

**Table 2:** Distance-based methods of population structure analysis

Reference	Clustering	Tool/package
Gao at el.(2007) [44]	Ward’s minimum variance algorithm	AWclust [84]: R package
Bouaziz at el. (2012) [43]	Spectral clustering (GMM)	SHIPS [85]: R package
Neuditschko at el.(2012) [63]	Super paramagnetic clustering (SPC)	NETVIEW [86]: MATLAB
Limpiti at el. (2014) [64]	Neighbor-joining (NJ) tree-based clustering	iNJclust [87]: C++

#### Allele-sharing distance

For clustering genetic data, allele-sharing distance (ASD) is used to identify closely related and distantly related pairs of individuals. ASD is similar to identity by state (IBS) metric [25].

Given $$ x={\left({x}_{i,l}\right)}_{\begin{array}{c}1\le i\le n\\ {}1\le l\le p\end{array}} $$ is a *n* × *p* matrix where *n* is the number of individuals and *p* the number of SNPs. Each entry *x*_*i*, *l*_ corresponds to the genotype of individual *i* for the marker *l*. Then, the ASD between individuals *i* and *j* at locus *l*, denoted as *D*_*l*_(*i*, *j*), is defined as follows:

$$ {D}_l\left(i,j\right)=\left\{\begin{array}{cc}0& \mathrm{if}\ \mathrm{same}\ \mathrm{genotype}\\ {}1& \kern1.75em \mathrm{if}\ \mathrm{one}\ \mathrm{common}\ \mathrm{allele}\\ {}2& \kern1.25em \mathrm{if}\ \mathrm{no}\ \mathrm{common}\ \mathrm{allele}\end{array}\right. $$

Therefore, the total distance between individuals *i* and *j* can be calculated as:

$$ D\left(i,j\right)=\frac{1}{p}\sum \limits_{l=1}^p\left({D}_l\left(i,j\right)\right)\kern3.5em \mathrm{for}\ \mathrm{each}\ i\ \mathrm{and}\ j\ \epsilon\ \left[1,n\right] $$

or as

$$ D\left(i,j\right)=\frac{1}{p}\sum \limits_{l=1}^p\left(\left|{x}_{i,l}-{x}_{j,l}\right|\right)\kern2.6em \mathrm{for}\ \mathrm{each}\ i\ \mathrm{and}\ j\ \epsilon\ \left[1,n\right] $$

where *x*_*i*, *l*_, *x*_*j*, *l*_ are the individuals’ genotypes, coded as 0, 1, or 2 according to the number of reference alleles present at the locus *l*. The closer the pair of individuals are, genetically, the smaller the value of *D*(*i*, *j*).

Using the function *D*(*i*, *j*) to quantify the distance between each pair of individuals *i* and *j*, a distance matrix can be formed by combining the information for all pairs of individuals. The distance matrix, $$ ={\left({D}_{i,j}\right)}_{\begin{array}{c}1\le i\le n\\ {}1\le j\le n\end{array}} $$, is a squared matrix of *n* × *n*, where *n* is the number of individuals.

Based on ASD, a similarity measure can be inferred to measure the similarity between individuals *i* and *j* at locus *l*, denoted as *S*_*l*_(*i*, *j*), where:

$$ S\left(i,j\right)=\frac{1}{p}\sum \limits_{l=1}^p\left(2-\left|{x}_{il}-{x}_{jl}\right|\right)\kern2em \mathrm{for}\ \mathrm{each}\ i\ \mathrm{and}\ j\ \epsilon\ \left[1,n\right] $$

#### Clustering based on ASD

Distance-based clustering methods use the ASD matrix as an input to group individuals into populations. AWclust, SHIPS, NETVIEW, and iNJclust all distance-based clustering methods, are summarized in Table 2.

AWclust [42, 44] is a distance-based population structure exploration method. The first step of AWclust is to construct the ASD matrix between all pairs of individuals in the sample. The second step is to apply hierarchical clustering to infer clusters of individuals from the ASD matrix using Ward’s minimum variance algorithm [65, 66]. AWclust uses gap statistics [56] to select the optimal number of subpopulations *K*. The employment of gap statistics is computationally intensive as it involves an iterative statistical inference process [67]. To deal with the slow speed of calculating gap statistics, AWclust limits the number of inferred *K* to be 16 at maximum [67]. The execution of AWclust slows down dramatically when using a larger number of SNPs due to the increase in the size of the ASD matrix [67]. Deejai et al. [67] found that AWclust performs well only with a small number of SNP markers and in individuals with low diversity (i.e., the number of inferred subpopulations *K* is small), and thus, it is not suitable for performing large-scale population genetic analysis. The application of AWclust on HapMap project phase 1 [18] provided good results. It successfully differentiated the four ethnic populations in the dataset: African, European, Han Chinese, and Japanese individuals [44].

SHIPS [43, 68], or Spectral Hierarchical clustering for the Inference of Population Structure, is a distance-based method for inferring the structure of populations from genetic data. SHIPS applies a divisive strategy of hierarchical clustering followed by a pruning procedure to investigate population structure progressively. SHIPS constructs a binary tree to represent the substructure of a population using spectral clustering. Spectral clustering is applied to a pairwise distance matrix to divide a population into two subpopulations, and this is iterated for each of the two subpopulations. ASD is used within SHIPS; however, SHIPS can be used with any similarity matrix. SHIPS applies a pruning procedure along with gap statistics to determine the optimal number of subpopulations. A pruning procedure provides all possible clustering results. Thus, it allows a fast calculation of the gap statistics that requires all the clustering results of specified numbers of clusters. Moreover, because calculating gap statistics is time consuming, SHIPS applies a version of gap statistics that is less precise but has better experimental performance in estimating the optimal *K*. Experiments have involved applying SHIPS on two datasets: HapMap project phase 3 [19] and Pan-Asian [22]. These experiments have shown that SHIPS can accurately assign individuals to clusters with relatively low computational cost and estimate the number of clusters as well [43, 68]. In addition, SHIPS is quite robust such that several applications of SHIPS algorithm on the same dataset produce the same clustering result.

NETVIEW [63] is an analysis pipeline that combines a network-based clustering method with a visualization tool to infer fine-scale population structure. NETVIEW is composed of three key steps: distance matrix calculation, network construction and clustering, and network-based visualization. NETVIEW first calculates the ASD matrix that represents the relationships between all individuals in the dataset. Then, the ASD matrix is used to construct a population network using super paramagnetic clustering (SPC) [69]. In this network, nodes represent individuals, edges represent the relationship between a pair of individuals, and the thickness of edges represents the genetic distance. SPC is based on computing the *K*-nearest neighborhood to produce a cluster relationship matrix and a hierarchical tree of clusters. Specifically, SPC is implemented as Sorting Points Into Neighborhood (SPIN) [69, 70], which employs the Potts Hamiltonian model [71] to identify the number and size of clusters, known as cluster stability. The problem with SPC is how to specify the number of the nearest neighborhood an individual can have. Based on this number, NETVIEW produces clusters at optimal thresholds of genetic distance. The result of this algorithm provides a hierarchical clustering of individuals. However, NETVIEW uses a network-based visualization to present the population structure at a very fine scale, where highly interconnected individuals identify subpopulations. The empirical study in [63] involved applying NETVIEW on Human and Bovine HapMap datasets. The study demonstrated that NETVIEW could assign individuals to their corresponding subpopulations effectively and showed the genetic relatedness of individuals within their populations at a very fine scale.

iNJclust [64], or iterative Neighbor-Joining tree clustering, is an iterative application of graph-based clustering on a neighbor-joining (NJ) tree. The algorithm starts by computing the ASD matrix from the data. Then, an NJ tree is constructed based on the ASD matrix. Next, the algorithm performs a graph-based clustering to bisect the NJ tree into two subtrees. For each subtree, a new NJ tree is constructed based on the ASD matrix that contains only individuals within that subtree. The process of bisecting the NJ trees to create new subtrees is iterated until all subtrees become homogenous. The algorithm determines whether the cluster is homogeneous based on the fixation index. The fixation index (F_ST_) is a measure of genetic population substructure used to examine the overall genetic divergence among subpopulations [72]. The construction of the NJ tree starts with all individuals as the leaf nodes. Then, the pair of nodes that are nearest to each other are merged. The merging process is repeated until all nodes are merged into the tree. The distance between nodes is measured using the minimum evolution criteria [73] based on the ASD. For NJ tree clustering, the NJ tree is split into two subtrees by cutting the edge between the two nodes with the longest length. iNJclust assigns the individuals into populations and estimates the optimal number of populations. The clustering result of iNJclust is a binary tree, where each leaf node represents a population of a set of individuals, and the tree structure represents the relationships between populations. The experimental results of applying iNJclust on real and simulated data have indicated that iNJclust yields a reasonable estimation of the number of populations, a robust assignment of individuals, and a meaningful representation of relationships among populations with the binary tree [64].

## Selection of informative markers

Given that a large number of genetic markers can be used to infer population structure, reducing the number of markers is often desirable for efficient structure identification. In such settings, selecting ancestry informative markers (AIMs) aims to identify the minimum set of markers required to derive population structure and to reduce the genotyping cost. Selecting informative markers can be accomplished by using supervised or unsupervised methods. Supervised methods rely on prior knowledge of the ancestry of the individuals.

Informativeness for assignment (*I*_*n*_) [74] is a supervised measure that computes mutual information based on allele frequencies and relies on self-reported ancestry information from individuals. In contrast, PCAIM [15] is an unsupervised algorithm proposed to identify a set of informative markers that captures the structure of a population. It does not demand prior information about the ancestry/origin of individuals. The PCAIM algorithm applies PCA to determine markers that are correlated with the significant principal components and then assigns a score to each marker. Then, the algorithm returns the top scoring markers that correlate well with the top few eigenvectors. The algorithm is efficient in selecting the informative markers. It is computationally fast and suitable for large datasets.

The performance of *I*_*n*_ and PCAIM in selecting informative markers has been evaluated in [15] and was found to attain comparable results; in addition, a considerable overlap was found between the selected markers. The overlapping was expected since PCAIM ranks markers based on how well they can reproduce the structure of the dataset, whereas *I*_*n*_ determines which markers are most likely to be associated with major clusters in the dataset. Therefore, PCAIM selects either the same markers or markers that are in high linkage disequilibrium (LD) with markers selected using the *I*_*n*_ measure.

The selection of informative markers could potentially suffer from redundant markers. Typically, redundancy exists due to the correlation among markers that are in the LD region. To select a minimal set of informative markers, a redundancy removal step should be applied after the initial markers selection step to avoid redundancy and determine the final set of AIMs.

In the literature, two different methods have been proposed to filter out redundant markers. The first method deals with the problem as a Column Subset Selection Problem, which is a well-known problem in linear algebra [75]. In [75], the algorithm Greedy QR [76, 77] is employed to select the minimally correlated subset of markers. The algorithm essentially works as an iterative process to pick up the uncorrelated markers. This algorithm has an implementation in MATLAB, and it can run efficiently in a shorter amount of time using thousands of markers. On the other hand, the redundancy removal problem can be resolved via the clustering technique. In particular, a clustering-based strategy was employed in [14] to minimize the number of markers to the most informative and uncorrelated ones, which was inspired by [78] in data analysis. In simpler terms, the strategy applies a clustering technique to cluster markers into *K* clusters and then returns one representative marker for each cluster. In [14], the Cluto toolkit [79] was used with default parameters for clustering using a cosine similarity matrix. The advantage of applying clustering to identify redundant markers is that it returns *K* lists of markers. Within each list, the markers are interchangeable, thus providing some flexibility in choosing any informative marker that falls into the same cluster. In contrast, the first method just returns one set of non-redundant markers. Although the two approaches of redundancy removal had comparable performance, clustering was slightly more accurate but was five times slower than the first method [14].

## Discussion

Nonparametric approaches are increasingly being used to reveal population structure because of their great advantages of efficiency in handling high-dimensional genetic datasets [74]. Due to the high dimensionality of genetic data, it is imperative to reduce the dimensions of the data before clustering. In the literature of population structure analysis, PCA is employed as a dimension reduction technique for two purposes. The first purpose is feature extraction, where PCA is applied to transform the data to low-dimensional space where clustering will be performed. The second purpose is feature selection, where PCA is applied to select the informative genetic markers. To accomplish this, PCA is applied to a covariance matrix of genetic markers, and then the genetic markers that are well correlated with significant principal components are selected.

PCA is considered computationally efficient and performs well in detecting the genetic structure of populations. However, it is also argued that PCA not be efficient when used with correlated markers that naturally arise in any genetic data, especially in densely genotyped data. The problem is that a large number of redundant and correlated markers may mask the real structure of data. In practice, with large genotype data, there are linked markers due to linkage disequilibrium (LD) [28], which is considered dependent and redundant, and this may seriously distort the results of PCA. Moreover, dimension reduction methods, like PCA, consider the complete markers of the dataset to produce only one subspace, in which the clustering can then be performed. However, an issue would arise when the correlation between markers or the relevance of markers are significant for some clusters (i.e., populations) but not for complete datasets. Consequently, this issue can be resolved by subspace clustering. Subspace clustering computes multiple subspaces, where a different set of features is selected for each subspace. Then, individuals are clustered differently in each subspace according to the relevance of markers to describe those individuals. Subspace clustering may be a significant solution, inferring the population structure at a very fine scale.

Many distance-based methods have been developed to resolve the problem of clustering individuals into subpopulations. These methods have utilized different clustering techniques that required a matrix of pairwise distance/similarity between individuals. Allele-sharing distance (ASD) is widely used for this purpose. In [80], it is shown that the ASD between individuals from different subpopulations is always larger than that of individuals from the same subpopulations. Moreover, calculating the ASD for many SNP markers allows differentiation of the populations through the accumulated effect of SNP loci. However, distance assessment using ASD between individuals becomes increasingly meaningless as dimensionality increases. As with increasing the number of SNPs, the distances of the individual to its similar individuals and dissimilar individuals tend to be almost the same. Individuals appear almost alike because of correlated SNPs, which are considered “redundant,” while ASD treats each marker independently. Therefore, the identification of correlated markers might improve the inference of population structure from high-dimensional genetic data. Filtering those markers before calculating ASD could contribute to more accurate clustering results, as achieved within HiClust-ipPCA [58].

The clustering techniques used to identify the population genetic substructure can be categorized into partitional clustering and hierarchical clustering. Partitional clustering produces a flat clustering which divides the data into a pre-specified number of clusters *K* (e.g., K-means [81], DBMS [40], Lee’s [52]). In contrast, hierarchical clustering produces a hierarchy of clusters (e.g., AWclust [44], SHIPS [43], NETVIEW [63], ipPCA [11, 16, 58], iNJclust [64]). Hierarchical clustering is preferable over partitional clustering in the context of population structure analysis. This is because it produces multiple nested partitions instead of one partition, which allows the choice of different partitions according to the desired level of similarity. Most importantly, a fine-scale population substructure can be obtained using hierarchical clustering because of the clustering’s ability to capture data at different levels of granularity.

A major challenge in population structure analysis is the estimation of the optimal number of subpopulations (i.e., clusters). Gap statistics [56] have often been applied to determine the optimal number of clusters. However, gap statistics is computationally intensive and impractical for highly structured genetic datasets that comprise a large number of clusters. Some clustering methods can implicitly determine the optimal number of clusters—for instance, ipPCA [11, 16, 58], where the number of clusters is represented by the number of leaf nodes of the binary tree constructed by iterative applications of PCA. However, determining the number of populations as a single number is not practical and may have no biological meaning when there are hierarchical levels of population structure (i.e., subpopulations within populations). Furthermore, the researcher must be able to control the level of granularity to uncover the substructure of the population. Overall, these provide insights into the importance of presenting the clustering result as a hierarchy whereby the researcher can visually determine the optimal level of separation from the number of major clusters in the dendrogram. The dendrogram serves as a visual means for both understanding the structure of the data and selecting a reasonable number of clusters.

## Conclusion

The analysis of population structure is used to obtain a clear insight into the underlying genetic population substructure and is a crucial prerequisite for any analysis of genetic data, such as genome-wide association studies, to eventually reduce false positive rates, and for forensics to develop reference panels that provide information on an individual’s ancestry. Single nucleotide polymorphisms (SNPs) are the most widely used markers to study the variation of DNA sequences between populations. Data preprocessing is a necessary step to assess the quality of the data before analysis, including the assessment of the call rates of both SNPs and individuals, minor allele frequency, and relatedness between individuals, where a threshold is set to eliminate SNPs/individuals that do not meet that threshold. Additionally, the selection of ancestry informative markers (AIMs), which are the minimal set of markers required to derive population structure, is considered important in preprocessing to improve the accuracy of clustering results.

After preprocessing, several analysis methods, including parametric and nonparametric, are used. Parametric approaches are impractical for large datasets because of their intensive computational cost, genetic assumptions that must be held, and sensitivity to sample size. In contrast, nonparametric approaches have the advantage of efficient computational cost with no modeling assumption requirements, making them more viable than parametric approaches for analyzing large datasets. Nonparametric approaches can be categorized into dimension reduction-based and distance-based methods. On the one hand, dimension reduction techniques are used to reduce the dimensions of genetic markers before conducting a clustering. The most used dimension reduction technique is principal components analysis (PCA), as it is implemented in EIGENSTRAT\smartpca. On the other hand, distance-based methods include computing dis/similarity matrices of the data where the clustering method is applied, such as AWclust, SHIPS, NETVIEW, and iNJclust. In these methods, similarity is measured using allele-sharing distance (ASD). ASD is a measure to determine how genetically close each pair of individuals is.

All in all, as evident in the challenges introduced by the ever-growing sizes and complexity of genetic datasets, accurate and efficient analysis methods are increasingly desirable to take full advantage of these available genetic datasets.

## Abbreviations

AIMs: Ancestry informative markers
ASD: Allele-sharing distance
AWclust: Allele-sharing distance and Ward’s minimum variance hierarchical clustering
DBMC: Density-based mean clustering
GWAS: Genome-wide association studies
HWE: Hardy-Weinberg equilibrium
iNJclust: Iterative neighbor-joining tree clustering
ipPCA: Iterative pruning PCA
LD: Linkage disequilibrium
MDS: Multi-dimensional scaling
NJ: Neighbor-joining
PCA: Principal component analysis
SHIPS: Spectral Hierarchical clustering for the Inference of Population Structure
SVD: Singular value decomposition
TW: Tracy-Widom

## References

[1] Lawson DJ, Falush D (2012). Population identification using genetic data. Annu Rev Genomics Hum Genet.

[2] Pritchard JK, Donnelly P (2001). Case-control studies of association in structured or admixed populations. Theor Popul Biol.

[3] Hoggart CJ, Parra EJ, Shriver MD, Bonilla C, Kittles RA, Clayton DG, McKeigue PM (2003). Control of confounding of genetic associations in stratified populations. Am J Hum Genet.

[4] Marchini J, Cardon LR, Phillips MS, Donnelly P (2004). The effects of human population structure on large genetic association studies. Nat Genet.

[5] Helgason A, Yngvadóttir B, Hrafnkelsson B, Gulcher J, Stefánsson K (2005). An Icelandic example of the impact of population structure on association studies. Nat Genet.

[6] Ziv E, Burchard EG (2003). Human population structure and genetic association studies. Pharmacogenomics.

[7] Freedman ML, Reich D, Penney KL, McDonald GJ, Mignault AA, Patterson N, Gabriel SB, Topol EJ, Smoller JW, Pato CN (2004). Assessing the impact of population stratification on genetic association studies. Nat Genet.

[8] Price AL, Zaitlen NA, Reich D, Patterson N (2010). New approaches to population stratification in genome-wide association studies. Nat Rev Genet.

[9] Kidd KK, Pakstis AJ, Speed WC, Grigorenko EL, Kajuna SL, Karoma NJ, Kungulilo S, Kim J-J, Lu R-B, Odunsi A (2006). Developing a SNP panel for forensic identification of individuals. Forensic Sci Int.

[10] Kidd KK, Speed WC, Pakstis AJ, Furtado MR, Fang R, Madbouly A, Maiers M, Middha M, Friedlaender FR, Kidd JR. Progress toward an efficient panel of SNPs for ancestry inference. Forensic Sci Int Genet. 2014;10:23–32. 10.1016/j.fsigen.2014.01.002 24508742

[11] Intarapanich A, Shaw PJ, Assawamakin A, Wangkumhang P, Ngamphiw C, Chaichoompu K, Piriyapongsa J, Tongsima S (2009). Iterative pruning PCA improves resolution of highly structured populations. BMC bioinformatics.

[12] Pritchard JK, Stephens M, Donnelly P (2000). Inference of population structure using multilocus genotype data. Genetics.

[13] Liu Y, Nyunoya T, Leng S, Belinsky SA, Tesfaigzi Y, Bruse S. Softwares and methods for estimating genetic ancestry in human populations. Hum Genomics. 2013;7(1):1. 10.1186/1479-7364-7-1 PMC3542037 23289408

[14] Paschou P, Lewis J, Javed A, Drineas P (2010). Ancestry informative markers for fine-scale individual assignment to worldwide populations. J Med Genet.

[15] Paschou P, Ziv E, Burchard EG, Choudhry S, Rodriguez-Cintron W, Mahoney MW, Drineas P (2007). PCA-correlated SNPs for structure identification in worldwide human populations. PLoS Genet.

[16] Limpiti T, Intarapanich A, Assawamakin A, Shaw PJ, Wangkumhang P, Piriyapongsa J, Ngamphiw C, Tongsima S (2011). Study of large and highly stratified population datasets by combining iterative pruning principal component analysis and structure. BMC bioinformatics.

[17] Brookes AJ (1999). The essence of SNPs. Gene.

[18] The International HapMap C (2005). A haplotype map of the human genome. Nature.

[19] Pemberton TJ, Wang C, Li JZ, Rosenberg NA (2010). Inference of unexpected genetic relatedness among individuals in HapMap phase III. Am J Hum Genet.

[20] Consortium IH (2007). A second generation human haplotype map of over 3.1 million SNPs. Nature.

[21] Consortium GP (2012). An integrated map of genetic variation from 1,092 human genomes. Nature.

[22] Ngamphiw C, Assawamakin A, Xu S, Shaw PJ, Yang JO, Ghang H, Bhak J, Liu E, Tongsima S, Consortium HP-AS (2011). PanSNPdb: the Pan-Asian SNP genotyping database. PLoS One.

[23] Laurie CC, Doheny KF, Mirel DB, Pugh EW, Bierut LJ, Bhangale T, Boehm F, Caporaso NE, Cornelis MC, Edenberg HJ (2010). Quality control and quality assurance in genotypic data for genome-wide association studies. Genet Epidemiol.

[24] Purcell S, Neale B, Todd-Brown K, Thomas L, Ferreira Manuel AR, Bender D, Maller J, Sklar P, de Bakker Paul IW, Daly Mark J, Sham Pak C (2007). PLINK: a tool set for whole-genome association and population-based linkage analyses. Am J Hum Genet.

[25] Stevens EL, Heckenberg G, Roberson ED, Baugher JD, Downey TJ, Pevsner J (2011). Inference of relationships in population data using identity-by-descent and identity-by-state. PLoS Genet.

[26] Manichaikul A, Mychaleckyj JC, Rich SS, Daly K, Sale M, Chen W-M (2010). Robust relationship inference in genome-wide association studies. Bioinformatics.

[27] Purcell S, Sham P (2005). Properties of structured association approaches to detecting population stratification. Hum Hered.

[28] Reich DE, Cargill M, Bolk S, Ireland J, Sabeti PC, Richter DJ, Lavery T, Kouyoumjian R, Farhadian SF, Ward R, Lander ES (2001). Linkage disequilibrium in the human genome. Nature.

[29] Porras-Hurtado L, Ruiz Y, Santos C, Phillips C, Carracedo Á, Lareu MV. An overview of STRUCTURE: applications, parameter settings, and supporting software. Front Genet. 2013;4:98. 10.3389/fgene.2013.00098 PMC3665925 23755071

[30] Alexander DH, Lange K (2011). Enhancements to the ADMIXTURE algorithm for individual ancestry estimation. BMC bioinformatics.

[31] Dawson KJ, Belkhir K (2001). A Bayesian approach to the identification of panmictic populations and the assignment of individuals. Genet Res.

[32] Corander J, Waldmann P, Sillanpää MJ (2003). Bayesian analysis of genetic differentiation between populations. Genetics.

[33] Corander J, Waldmann P, Marttinen P, Sillanpää MJ (2004). BAPS 2: enhanced possibilities for the analysis of genetic population structure. Bioinformatics.

[34] Guillot G, Mortier F, Estoup A (2005). GENELAND: a computer package for landscape genetics. Mol Ecol Notes.

[35] Tang H, Peng J, Wang P, Risch NJ (2005). Estimation of individual admixture: analytical and study design considerations. Genet Epidemiol.

[36] Alexander DH, Novembre J, Lange K (2009). Fast model-based estimation of ancestry in unrelated individuals. Genome Res.

[37] Wu B, Liu N, Zhao H (2006). PSMIX: an R package for population structure inference via maximum likelihood method. BMC bioinformatics.

[38] Raj A, Stephens M, Pritchard JK. fastSTRUCTURE: variational inference of population structure in large SNP datasets. Genetics. 2014;197(2):573–89. 10.1534/genetics.114.164350 PMC4063916 24700103

[39] Price AL, Patterson NJ, Plenge RM, Weinblatt ME, Shadick NA, Reich D (2006). Principal components analysis corrects for stratification in genome-wide association studies. Nat Genet.

[40] Liu N, Zhao H (2006). A non-parametric approach to population structure inference using multilocus genotypes. Human genomics.

[41] Patterson N, Price AL, Reich D (2006). Population structure and eigenanalysis. PLoS Genet.

[42] Gao X, Starmer JD (2008). AWclust: point-and-click software for non-parametric population structure analysis. BMC bioinformatics.

[43] Bouaziz M, Paccard C, Guedj M, Ambroise C (2012). SHIPS: spectral hierarchical clustering for the inference of population structure in genetic studies. PLoS One.

[44] Gao X, Starmer J (2007). Human population structure detection via multilocus genotype clustering. BMC Genet.

[45] Bryc K, Auton A, Nelson MR, Oksenberg JR, Hauser SL, Williams S, Froment A, Bodo J-M, Wambebe C, Tishkoff SA (2010). Genome-wide patterns of population structure and admixture in West Africans and African Americans. Proc Natl Acad Sci.

[46] Bryc K, Velez C, Karafet T, Moreno-Estrada A, Reynolds A, Auton A, Hammer M, Bustamante CD, Ostrer H (2010). Genome-wide patterns of population structure and admixture among Hispanic/Latino populations. Proc Natl Acad Sci.

[47] Novembre J, Stephens M (2008). Interpreting principal component analyses of spatial population genetic variation. Nat Genet.

[48] Zheng X, Levine D, Shen J, Gogarten SM, Laurie C, Weir BS (2012). A high-performance computing toolset for relatedness and principal component analysis of SNP data. Bioinformatics.

[49] McVean G (2009). A genealogical interpretation of principal components analysis. PLoS Genet.

[50] Tracy CA, Widom H (1994). Level-spacing distributions and the airy kernel. Commun Math Phys.

[51] Johnstone IM. On the distribution of the largest eigenvalue in principal components analysis. Ann Stat. 2001;29(2):295–327.

[52] Lee C, Abdool A, Huang C-H: PCA-based population structure inference with generic clustering algorithms**.** BMC bioinformatics 2009, 10**:**S73. 10.1186/1471-2105-10-S1-S73 PMC2648762 19208178

[53] Hartigan JA, Wong MA. Algorithm AS 136: a k-means clustering algorithm. Appl Stat. 1979:100–8.

[54] Fraley C, Raftery AE (2003). Enhanced model-based clustering, density estimation, and discriminant analysis software: MCLUST. J Classif.

[55] Ng AY, Jordan MI, Weiss Y. On spectral clustering: analysis and an algorithm. In: Proceedings of advances in neural information processing systems. Cambridge: MIT Press; 2001. p. 849–56.

[56] Tibshirani R, Walther G, Hastie T (2001). Estimating the number of clusters in a data set via the gap statistic. J Royal Stat Soc Series B (Statistical Methodology).

[57] Schwarz G (1978). Estimating the dimension of a model. Ann Stat.

[58] Amornbunchornvej C, Limpiti T, Assawamakin A, Intarapanich A, Tongsima S: Improved iterative pruning principal component analysis with graph-theoretic hierarchical clustering**.** In 9th international conference on electrical engineering/electronics, computer, telecommunications and information technology; 16–18 2012. 2012: 1–4.

[59] Luo J, Zhang Z: Using eigenvalue grads method to estimate the number of signal source**.** In 2000 5th International Conference on Signal Processing Proceedings; Beijing. IEEE; 2000: 223–225.

[60] Wall ME, Rechtsteiner A, Rocha LM (2003). Singular value decomposition and principal component analysis.

[61] Li M, Reilly C, Hanson T (2008). A semiparametric test to detect associations between quantitative traits and candidate genes in structured populations. Bioinformatics.

[62] Mountain JL, Cavalli-Sforza LL. Inference of human evolution through cladistic analysis of nuclear DNA restriction polymorphisms. Proc Natl Acad Sci. 1994;91(14):6515–19. 10.1073/pnas.91.14.6515 PMC44233 7912828

[63] Neuditschko M, Khatkar MS, Raadsma HW (2012). NetView: a high-definition network-visualization approach to detect fine-scale population structures from genome-wide patterns of variation. PLoS One.

[64] Limpiti T, Amornbunchornvej C, Intarapanich A, Assawamakin A, Tongsima S (2014). iNJclust: iterative neighbor-joining tree clustering framework for inferring population structure. IEEE/ACM Trans Comput Biol Bioinformatics.

[65] Ward Jr JH (1963). Hierarchical grouping to optimize an objective function. J Am Stat Assoc.

[66] Ward Jr JH, Hook ME. Application of an hierarchial grouping procedure to a problem of grouping profiles. Educ Psychol Meas. 1963;23(1):69–81.

[67] Deejai P, Assawamakin A, Wangkumhang P, Poomputsa K, Tongsima S: On assigning individuals from cryptic population structures to optimal predicted subpopulations: an empirical evaluation of non-parametric population structure analysis techniques. In Computational Systems-Biology and Bioinformatics. Berlin: Springer; 2010. p. 58–70.

[68] Bouaziz M: SHIPS: spectral hierarchical clustering for the inference of population structure**.** In Annals of Human Genetics*;* NJ,USA. WILEY-BLACKWELL; 2012: 413–413. 10.1371/journal.pone.0045685 PMC3470591 23077494

[69] Blatt M, Wiseman S, Domany E (1996). Superparamagnetic clustering of data. Phys Rev Lett.

[70] Tsafrir D, Tsafrir I, Ein-Dor L, Zuk O, Notterman DA, Domany E (2005). Sorting points into neighborhoods (SPIN): data analysis and visualization by ordering distance matrices. Bioinformatics.

[71] Tetko IV, Facius A, Ruepp A, Mewes H-W (2005). Super paramagnetic clustering of protein sequences. BMC Bioinformatics.

[72] Holsinger KE, Weir BS (2009). Genetics in geographically structured populations: defining, estimating and interpreting F ST. Nat Rev Genet.

[73] Gascuel O, Steel M (2006). Neighbor-joining revealed. Mol Biol Evol.

[74] Rosenberg NA, Li LM, Ward R, Pritchard JK (2003). Informativeness of genetic markers for inference of ancestry. Am J Hum Genet.

[75] Paschou P, Drineas P, Lewis J, Nievergelt CM, Nickerson DA, Smith JD, Ridker PM, Chasman DI, Krauss RM, Ziv E. Tracing sub-structure in the European American population with PCA-informative markers. PLoS Genet. 2008;4(7):e1000114. 10.1371/journal.pgen.1000114 PMC2537989 18797516

[76] Golub G (1965). Numerical methods for solving linear least squares problems. Numer Math.

[77] Gu M, Eisenstat SC (1996). Efficient algorithms for computing a strong rank-revealing QR factorization. SIAM J Sci Comput.

[78] Boutsidis C, Sun J, Anerousis N: Clustered subset selection and its applications on it service metrics**.** In Proceedings of the 17th ACM conference on Information and knowledge management. ACM; 2008: 599–608.

[79] Zhao Y, Karypis G: Evaluation of hierarchical clustering algorithms for document datasets**.** In Proceedings of the eleventh international conference on Information and knowledge management. ACM; 2002: 515–524.

[80] Gao X, Martin ER (2009). Using allele sharing distance for detecting human population stratification. Hum Hered.

[81] Jombart T, Devillard S, Balloux F (2010). Discriminant analysis of principal components: a new method for the analysis of genetically structured populations. BMC Genet.

[82] EIGENSTRAT/smartpca [http://www.hsph.harvard.edu/alkes-price/software/]. Accessed 20 Jan 2018.

[83] ipPCA [http://www4a.biotec.or.th/GI/tools/ippca]. Accessed 20 Jan 2018.

[84] AWclust [http://awclust.sourceforge.net/]. Accessed 20 Jan 2018.

[85] SHIPS [http://www.math-evry.cnrs.fr/logiciels/ships]. Accessed 20 Apr 2018.

[86] NETVIEW [http://sydney.edu.au/vetscience/reprogen/netview/]. Accessed 20 Jan 2018.

[87] iNJclust [http://www4a.biotec.or.th/GI/tools/injclust]. Accessed 20 Jan 2018.

